# The N-Terminus of Human Lactoferrin Displays Anti-biofilm Activity on *Candida parapsilosis* in Lumen Catheters

**DOI:** 10.3389/fmicb.2017.02218

**Published:** 2017-11-13

**Authors:** Roberta Fais, Mariagrazia Di Luca, Cosmeri Rizzato, Paola Morici, Daria Bottai, Arianna Tavanti, Antonella Lupetti

**Affiliations:** ^1^Department of Translational Research and of New Technologies in Medicine and Surgery, University of Pisa, Pisa, Italy; ^2^Berlin-Brandenburg Center for Regenerative Therapies, Charité – University Medicine Berlin, Berlin, Germany; ^3^National Enterprise for nanoScience and nanoTechnology, Istituto Nanoscienze-Consiglio Nazionale delle Ricerche and Scuola Normale Superiore, Pisa, Italy; ^4^Department of Biology, University of Pisa, Pisa, Italy

**Keywords:** *Candida parapsilosis*, biofilm, antimicrobial peptides, hLF 1-11, catheter-related infection

## Abstract

*Candida parapsilosis* is a major cause of hospital-acquired infection, often related to parenteral nutrition administered via catheters and hand colonization of health care workers, and its peculiar biofilm formation ability on plastic surfaces. The mortality rate of 30% points to the pressing need for new antifungal drugs. The present study aimed at analyzing the inhibitory activity of the N-terminal lactoferrin-derived peptide, further referred to as hLF 1-11, against biofilms produced by clinical isolates of *C. parapsilosis* characterized for their biofilm forming ability and fluconazole susceptibility. hLF 1-11 anti-biofilm activity was assessed in terms of reduction of biofilm biomass, metabolic activity, and observation of sessile cell morphology on polystyrene microtiter plates and using an *in vitro* model of catheter-associated *C. parapsilosis* biofilm production. Moreover, fluctuation in transcription levels of genes related to cell adhesion, hyphal development and extracellular matrix production upon peptide exposure were evaluated by quantitative real time RT-PCR. The results revealed that hLF 1-11 exhibits an inhibitory effect on biofilm formation by all the *C. parapsilosis* isolates tested, in a dose-dependent manner, regardless of their fluconazole susceptibility. In addition, hLF 1-11 induced a statistically significant dose-dependent reduction of preformed-biofilm cellular density and metabolic activity at high peptide concentrations only. Interestingly, when assessed in a catheter lumen, hLF 1-11 was able to induce a 2-log reduction of sessile cell viability at both the peptide concentrations used in RPMI diluted in NaPB. A more pronounced anti-biofilm effect was observed (3.5-log reduction) when a 10% glucose solution was used as experimental condition on both early and preformed *C. parapsilosis* biofilm. Quantitative real time RT-PCR experiments confirmed that hLF 1-11 down-regulates key biofilm related genes. The overall findings suggest hLF 1-11 as a promising candidate for the prevention of *C. parapsilosis* biofilm formation and to treatment of mature catheter-related *C. parapsilosis* biofilm formation.

## Introduction

*Candida* species are the fourth most common isolated microorganism from blood cultures (Sardi et al., 2013). Among these yeasts, although *Candida albicans* still predominates, infections due to non-*albicans* species have emerged over the past two decades (van Asbeck et al., 2009; Pfaller et al., 2010; Cleveland et al., 2012). In this context, the rise of *Candida parapsilosis*, which is now the second or third most common yeast species recovered from blood cultures in different regions of Europe (Marra et al., 2011; Silva et al., 2012), ranging from 20% in adults to 44% in premature newborns (van Asbeck et al., 2009; Bassetti et al., 2011, 2013; Ballot et al., 2013), mainly correlates with increased use of caspofungin and voriconazole (Forrest et al., 2008). The increased incidence of *C. parapsilosis* candidemia is also associated with therapeutic regimen-related features (e.g., extended hospital stay) as well as with microorganism-related characteristics, such as the selective adherence to prosthetic materials, biofilm formation ability on plastic surfaces (Pammi et al., 2013), and proliferation in high concentration of glucose and lipids (Branchini et al., 1994). *C. parapsilosis* ability to form biofilms on the surface and lumen of catheters is highly strain dependent (Kuhn et al., 2002). *C. parapsilosis* is known to colonize the hands of health care workers, thus emphasizing the need of hand hygiene and proper catheter care (Lupetti et al., 2002; Bonassoli et al., 2005; Pammi et al., 2013; Barbedo et al., 2015). Since the high glucose environment associated with parenteral nutrition contributes to biofilm formation (Dotis et al., 2012), *C. parapsilosis* is recognized as the major cause of hospital-acquired infection due to *Candida* spp. (Douglas, 2003). Biofilm formation leads to a structured microbial community that is attached to a biotic or abiotic surface and embedded in an exopolymeric extracellular matrix (Chandra et al., 2001; Donlan and Costerton, 2002; Douglas, 2003), which prevents the entrance of most commonly used antifungal agents. As microbes embedded in biofilms may be resistant to antifungals (Tumbarello et al., 2007; van Asbeck et al., 2009), these *C. parapsilosis* infections are often associated with a crude mortality rate of 30% (Maganti et al., 2011; Nucci et al., 2013; Parmeland et al., 2013; Magobo et al., 2017).

This points to a pressing need for new antifungal agents, e.g., antimicrobial proteins or peptides (Zasloff, 2002; Hancock and Sahl, 2006). Antimicrobial peptides have emerged as an attractive target area from which to source new antibiofilm solutions (Moreno et al., 2017). A collection of biofilm-active antimicrobial peptides, with their effects on medically relevant species, including Candida spp, can be found in the open-access “BaAMPs” database (www.baamps.it; Di Luca et al., 2014). The 77 kD antimicrobial protein lactoferrin is part of the innate defense system and is provided to newborns during breast-feeding. It is an iron-binding glycoprotein synthesized by mucosal gland epithelial cells and neutrophils (Nuijens et al., 1996) and released by the latter cells in response to inflammatory stimuli (Brock, 1995; Lonnerdal and Iyer, 1995). Its role in the innate defense system seems to be related to the release of peptides with wide spectrum microbicidal activity. Indeed, when human lactoferrin (hLF) is subjected to pepsinolysis it releases the antimicrobial peptide lactoferricin H (residues 1 to 47) (Bellamy et al., 1992), which contains two cationic domains (residues 2 to 5 and residues 28 to 31). A robust body of evidence indicates that the synthetic peptide corresponding to residues 1-11 of hLF (GRRRRSVQWCA; molecular mass, 1374.6 Da), reported as hLF 1-11, that includes the first cationic domain of hLF (Chapple et al., 1998), is highly effective in killing yeasts in *in vitro* and *in vivo* experiments (Lupetti et al., 2000, 2002, 2003, 2007) and a recent study has demonstrated that the peptide is also able to inhibit biofilm formation by *C. albicans* mainly at early stages (Morici et al., 2016).

The present study was undertaken (i) to evaluate the potential inhibitory activity of hLF 1-11 against biofilm formation of clinical isolates of *C. parapsilosis* characterized for their biofilm forming ability and fluconazole susceptibility, (ii) to gain more insight into the molecular mechanism(s) underlying the hLF 1-11-induced antibiofilm activity, and (iii) to confirm the anti-biofilm effect exerted by the peptide in an *in vitro* model of catheter infection.

## Materials and Methods

### Strains

*Candida parapsilosis* strains used in this study are listed in **Table 1**. *C. parapsilosis* strains were stored in YPD broth (Yeast Peptone Dextrose, Difco BD, Milan, Italy) supplemented with 40% glycerol at -20 and -80°C. Isolates were subcultured at 30°C on YPD agar plates, and kept at 4°C until completion of the study. Liquid cultures were prepared in YPD broth starting from a single colony. Following overnight incubation at 30°C, fungal cells were washed twice in sodium phosphate buffer (NaPB, pH 7), and diluted at the desired concentration after cell counting using a Bürker chamber.

**Table 1:** Details of the *Candida parapsilosis* strains used in this study.

Strain	Source	Body site	Biofilm formation	Fluconazole susceptibility (mg/L)
CP 7	A.O.U.P. Pisa, Italy	Skin	Strong (Tavanti et al., 2010)	0.5
CP 577	A.O.U.P. Pisa, Italy	Urine	Medium (This study)	1
CP 558	Rosario, Argentina	Abscess	Medium (Tavanti et al., 2010)	16
ATCC 22019	Reference strain, United States	/	Weak (Tavanti et al., 2010)	1
CP 508^∗^	UCD University, Ireland	Laboratory strain	Weak (Ding and Butler, 2007)	1

*^∗^The strain CP 508, lacking both copies of *BCR1* gene (*Δbcr1/Δbcr1*) (Ding and Butler, 2007), was kindly provided by Geraldine Butler (UCD University, Dublin, Ireland). A.O.U.P., Azienda Ospedaliero-Universitaria Pisana.*

### Lactoferrin Peptide

The synthetic peptide corresponding to residues 1-11 of human lactoferrin was purified by Peptisyntha Inc. (Torrance, CA, United States). hLF 1-11 stocks were prepared in NaPB (0.1 M, pH 7) with 0.01% acetic acid (pH 3.7) at a final concentration of 10 mM and were stored at -20°C.

### Biofilm Formation Assay

Biofilm formation assays were performed as previously described for *C. albicans* (Morici et al., 2016). Briefly, *C. parapsilosis* yeast suspensions were prepared at 2 × 10^6^ cells/mL in four-fold diluted RPMI 1640 medium (supplemented with 2% glucose and MOPS, pH 7), and seeded in polystyrene, flat-bottomed, 96-well microtiter plates (100 μl/well). CP 7 and ATCC 22019 *C. parapsilosis* strains were included in each experiment as positive and negative control, respectively. Following incubation at 37°C for 24 h, non-adhered cells were removed by washing twice with phosphate buffered saline (PBS). Biofilm production was evaluated by measuring the total biofilm biomass and cellular metabolic activity. Biofilm biomass formed in the presence or absence of hLF 1-11 was measured (OD_λ490 nm_) using an automated plate reader (Model 550 Microplate Reader Bio-Rad, Milan, Italy). Background optical density was subtracted from the values measured in each well.

The cellular metabolic activity was evaluated by the XTT/menadione assay. XTT solution was prepared at 0.5 g/L in PBS buffer and mixed with a menadione solution dissolved in acetone at a final concentration of 1 μM. An aliquot of 100 μl XTT/menadione solution was inoculated into each well of a 96-well plate containing dry preformed biofilms and incubated in the dark at 37°C. Following a 2 h incubation, the supernatant (80 μL) was transferred into a 96-well plate to measure colorimetric changes at 490 nm, once background optical density was subtracted from each well. Strains were arbitrarily divided into strong, medium, and weak biofilm producers based on the optical density value, as previously described (Tavanti et al., 2010). Three independent experiments were performed, each in triplicate.

### Inhibition of Biofilm Formation

Biofilm formation assays were carried out as described above. hLF 1-11 was added to the yeast suspension at three different concentrations (44, 88, and 176 mg/L), in a final volume of 100 μL/well. Positive and negative controls (untreated fungal suspension and medium alone, respectively) were included in each experiment. After incubation at 37°C for 24 h, non-adhered cells were removed and the amount of biofilm produced in presence of hLF 1-11 was determined by measuring both the total biofilm density and the metabolic activity as indicated above.

### hLF 1-11 Activity on *C. parapsilosis* Pre-adhered Cells and Preformed Biofilm

*Candida parapsilosis* strains CP 7, CP 558 and CP 577 (1 × 10^6^ cells/mL in RPMI diluted in NaPB, as previously described) were incubated in 96-well plates for 24 h. These experimental conditions allowed the formation of mature biofilm. In addition, *C. parapsilosis* strain CP 7 was incubated for 1.5, 3, and 6 h to allow fungal cells to adhere to the well surface prior to peptide treatment. Following incubation, non-adhered cells were removed and 100 μL of hLF 1-11 solution in four-fold diluted RPMI in NaPB were added to each well at three different peptide concentrations (44, 88, 176 mg/L). Plates were further incubated for 24 h at 37°C and biofilm-related parameters (biomass and metabolic activity) were determined as described above. Three independent experiments were performed, each in triplicate.

### hLF 1-11 Activity on *C. parapsilosis* Cell Morphology in Biofilm

The morphology of sessile cells embedded in biofilm formed by different *C. parapsilosis* clinical isolates was visualized under an inverted microscope (Olympus IMT-2) at 400× magnification in a 96-well microtiter plate following 24 h of incubation at 37°C. Cell morphology of the strong biofilm producer strain (CP 7) was observed following co-incubation with hLF 1-11 for 24 h at 37°C, compared to untreated control.

### Transcriptional Analysis of *C. parapsilosis* Biofilm-Related Genes by qRT-PCR

Biofilm production assay was performed with strain CP 7 treated with hLF 1-11 44 mg/L in 6-well plates for 24 h at 37°C. Untreated CP 7 biofilm was used as a positive control. Null mutant CP 508 strain, lacking both copies of Cp*BCR1* gene (*bcr1Δ/bcr1Δ*) and impaired in biofilm production (Ding and Butler, 2007), was also included in the experiment set. After incubation, adhered cells were removed from the bottom of the wells by gentle scraping (2 wells/sample) and suspended in 1× PBS. Total RNA was extracted with the Nucleospin RNA Kit (Macherey Nagel, Duren, Germany) and stored at -80°C. The quality and quantity of the extracted RNA were determined spectrophotometrically. Total RNA (1 μg) was converted into cDNA with N6 random primers in a 20 μL reaction volume, using the Reverse Transcription System kit (Promega Inc., Madison, WI, United States). The expression of genes involved in the adhesion process [*CPAR2_404790 (CpALS6), CpALS7* (Bertini et al., 2016), *CPAR2_500660 (CpALS10); CPAR2_404770 (CpALS11), CPAR2_404780 (CpALS12*)], in biofilm maturation and morphogenesis (*CpACE2, CpCPH2, CpEFG1, CpBCR1*), and matrix production (*CpFSK1*) was determined by qRT-PCR. Primer sequences used for amplification of specific genes are shown in Supplementary Table S1. qRT-PCR mixtures contained 40 ng/6 μL cDNA, 10 μL SYBR Green Master Mix (Applied Biosystem, Life technologies, Monza, Italy) and 1 pMol/μL of each primer. The amplification was performed in 96-well plates on CFX96 Touch Real-Time PCR Detection System (BioRad Laboratories S.r.L., Milan, Italy) (95°C for 60 s, followed by 40 cycles of 95°C for 5 s, 60°C for 30 s). Actin (*ACT1*) was used as internal control. The transcription level of the selected genes was calculated using the formula of 2^-^^ΔΔC_T_^. Data were expressed as means ± standard error of mean (SEM) of three independent experiments, each performed in triplicate.

### hLF 1-11 Activity on *C. parapsilosis* Biofilm Formed on Catheter Lumen

The ability of hLF 1-11 to inhibit biofilm formation in *C. parapsilosis* was investigated in an *in vitro* model of catheter-associated *Candida* biofilm formation. Peripheral Teflon catheters (PVC) of 1 mm diameter and 32 mm length (Deltaven FEP, Deltamed SpA; Mantova, Italy) were used for biofilm formation assays and aseptically cut into 30 mm pieces. Control samples were represented by catheter pieces inoculated with 20 μL of *C. parapsilosis* CP 7 fungal cells (5 × 10^6^ cells/mL in RPMI 1:4 diluted in NaPB) using a micro-syringe. Control catheters were then placed into a 1.7 micro-centrifuge tube with 1.5 mL of diluted RPMI or an equal volume of 10% glucose solution. A set of catheters was inoculated with 20 μL of CP 7 and treated with hLF 1-11 44 mg/L or 88 mg/L. Peptide-treated catheters were then placed into a 1.7 micro-centrifuge tube containing 1.5 mL of the respective hLF 1-11 solution in RPMI diluted 1:4 in NaPB or in 10% glucose solution. The ability of hLF 1-11 to eradicate preformed biofilm formation in *C. parapsilosis* was also investigated. Briefly, strain CP 7 (5 × 10^6^ cells/mL in RPMI diluted in NaPB) was inoculated in catheter pieces as described above, and incubated for 24 h, to allow formation of mature biofilm. Following incubation, non-adhered cells were removed and 20 μL of hLF 1-11 solution (44 and 88 mg/L) in four-fold diluted RPMI in NaPB o 10% glucose solution were added to catheter, as described above. Peptide-treated catheters were then placed into a 1.7 micro-centrifuge tube containing 1.5 mL of the respective hLF 1-11 solution in RPMI diluted 1:4 in NaPB or in 10% glucose solution.

Following a 24 h incubation at 37°C, all colonized catheters were washed in PBS, internally and externally, and placed in new micro-centrifuge tubes containing fresh RPMI diluted 1:4 in NaPB or 10% glucose solution. Control and treated catheters were sonicated for 5 min and vortexed for further 5 min, to allow the complete detachment of biofilm embedded cells from the catheter lumen. Yeast suspension were then diluted in PBS and plated on SD agar and incubated at 37°C for 24 h, to quantify colony-forming units (CFUs). Three independent experiments were performed.

### Imaging of *in Vitro* Formed Biofilms by Confocal Laser Scanning Microscopy (CLSM)

Confocal laser scanning microscopy (CLSM) was used to evaluate the presence of biofilm formed in a catheter model of *C. parapsilosi*s infection, following hLF 1-11 treatment for 24 h at 37°C. Untreated biofilm grown in the catheter lumen for 24 h at 37°C served as positive control.

Briefly, control and peptide treated catheter pieces were inoculated with 20 μL of *C. parapsilosis* suspension containing 5 × 10^6^ CP 7 blastoconidia. Peptide-treated catheters were represented by catheter pieces inoculated with 20 μL of *C. parapsilosis* CP 7 fungal cells treated with hLF 1-11 44 mg/L; both catheter pieces were re-suspended in 1.5 mL four-fold diluted RPMI 1640 medium into a 1.7 micro-centrifuge tube. Following a 24 h incubation at 37°C, the infected catheters were washed in PBS, internally and externally and stained with 300 μL Syto9 (5 nM) and propidium iodide (30 nM) for observation with CLSM, as suggested by the producer (Thermo Fisher Scientific, Waltham, MA, United States). After staining, biofilms were washed three times with pure water and examined under a Leica CLSM. An argon laser was used to excite the fluorophores at a wavelength of 488 nm for Syto9. In a typical two-channel experiment, images were collected in sequential mode to eliminate emission cross talk or bleed-through between the various dyes. Only one experiment was performed and 10 images for each sample were collected for biofilm formation analysis.

### Statistical Analysis

Data were expressed as means ± SEM. Statistical analysis was performed with one-way ANOVA test, followed by the Tukey–Kramer *post hoc* test, using GraphPad Instat software (version 6.05 for Windows, La Jolla, CA, United States). Differences in gene expression levels by qRTPCR were analyzed by Student’s paired *t*-test (GraphPad prism, version 5.0). The level of significance was set at a *P-*value of ≤0.05.

## Results

### Biofilm Formation

A panel of *C. parapsilosis* strains (**Table 1**) was tested for the ability to form biofilm on polystyrene well plate. For each strain, the biofilm-forming ability was evaluated in terms of biofilm cellular density and metabolic activity of sessile cells (Supplementary Figure S1). The strain CP 7, used in the assay as positive control, was confirmed as strong biofilm producer (Tavanti et al., 2010). Strains CP 558 and CP 577 were classified as medium biofilm producers, while CP 508 and reference strain ATCC 22019 were used as weak biofilm producers. Interestingly, CP 508, a mutant strain lacking both copies of biofilm-associated *BCR1* gene and included in the test as negative control, showed indeed a low biofilm production as evaluated by biomass quantification but metabolic activity levels comparable to those observed for strain CP 7.

### hLF 1-11 Anti-biofilm Activity

**FIGURE 1:**
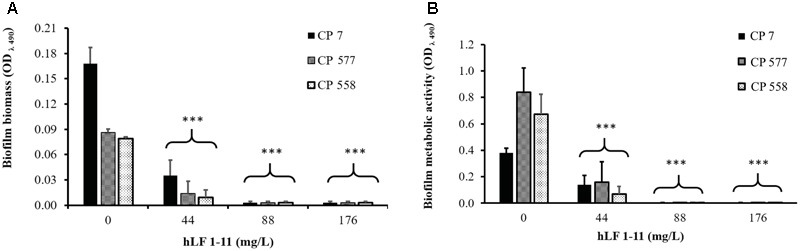
Effect of hLF 1-11 on biofilm formation by three *Candida parapsilosis* strains. Yeast cells were co-incubated with different concentrations of hLF 1-11 for 24 h at 37°C. The peptide activity was assessed in terms of biofilm biomass **(A)** and metabolic activity **(B)**. Data are expressed as means of three independent experiments ± SEM. ^∗∗∗^*P* ≤ 0.001.

The inhibitory activity of hLF 1-11 on biofilm formation was evaluated in three different clinical isolates (CP 7, CP 577, CP 558) exhibiting medium to high biofilm production ability. As shown in **Figure 1**, hLF 1-11 induced a marked reduction in biofilm cellular density and metabolic activity for the three strains tested at all the peptide concentrations used (44, 88, 176 mg/L) (*P* ≤ 0.001). For the strong biofilm producer strain (CP 7) the lowest peptide concentration induced a fivefold reduction in biofilm biomass, which was almost completely eradicated at higher hLF 1-11 concentrations. A similar trend could be observed for medium producer strains (**Figure 1A**). Accordingly, for all strains metabolic activity was not detectable at hLF 1-11 88 and 176 mg/L and was significantly reduced at the lowest peptide concentration used (**Figure 1B**).

### Effect of hLF 1-11 on *C. parapsilosis* Cell Morphology

**FIGURE 2:**
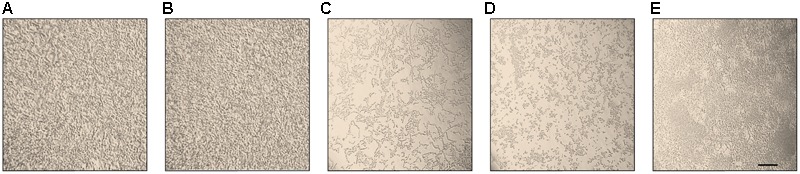
Inverted microscope images (400×) show biofilms produced by *C. parapsilosis* CP 7 **(A)**, CP 558 **(B)**, CP 577 **(C)**, CP 508 **(D)**, and ATCC 22019 **(E)** strains, following incubation for 24 h at 37°C in a polystyrene microtiter plate. Bar denotes 50 μm.

**FIGURE 3:**
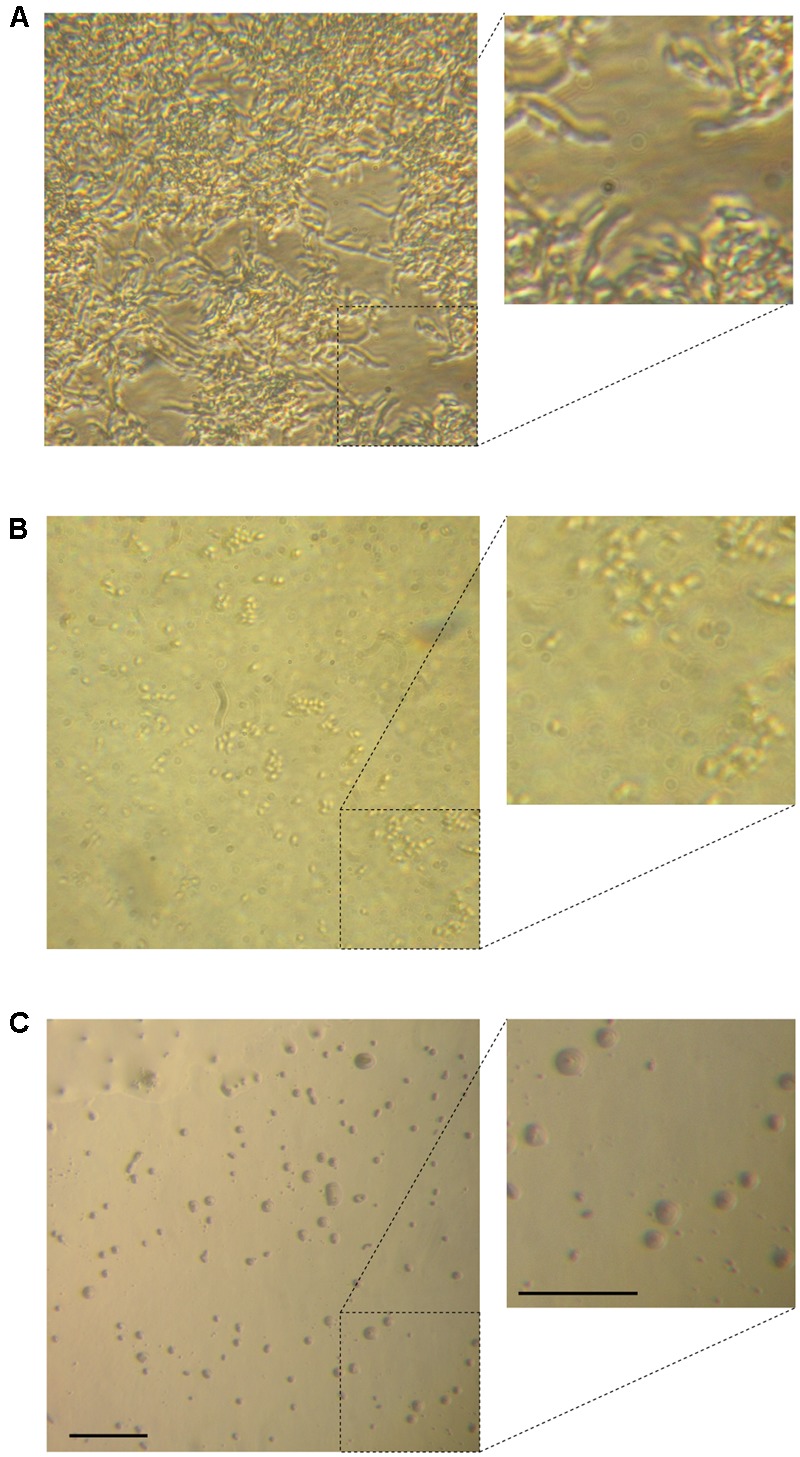
Inverted microscope images show sessile cell organization of strain CP 7 (strong biofilm producer) in the absence **(A)** and following co-incubation with 44 mg/L **(B)** or 88 mg/L **(C)** hLF 1-11 for 24 h at 37°C. Bars denote 50 μm.

The morphology of different *C. parapsilosis* strains included in this study was observed using an inverted microscope following 24 h growth at 37°C, under biofilm-inducing conditions. As depicted in **Figure 2**, strains CP 7, CP 577, CP 558 produced a thick multi-layer biofilm, in which both yeast and pseudohyphal cells could be observed. Conversely, strains CP 508 and ATCC 22019 produced a thin and patchy biofilm, where mainly pseudohyphae were visible (**Figure 2**). Notably, co-incubation of hLF 1-11 with *C. parapsilosis* CP 7 produced a complete inhibition of biofilm formation, with only few cells visible on the bottom of the well and predominantly in the yeast form (**Figure 3**).

### hLF 1-11 Activity on *C. parapsilosis* Pre-adhered Cells and on Preformed Biofilm

**FIGURE 4:**
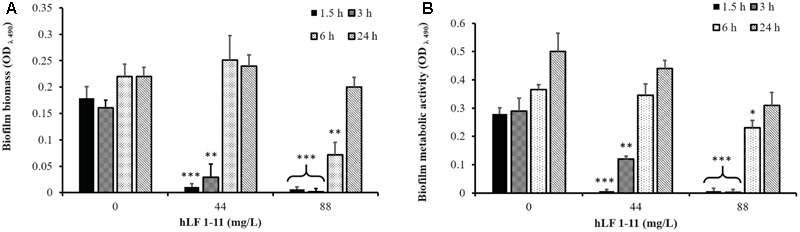
Effect of hLF 1-11 on pre-adhered cells of *C. parapsilosis.* Yeast cells were incubated at different time points (1.5, 3, 6, and 24 h) and then co-incubated with hLF 1-11 at two different concentrations for 24 h at 37°C. The peptide anti-biofilm activity was assessed in terms of reduction of biofilm biomass **(A)** and metabolic activity **(B)**. Data are expressed as means of three independent experiments ± SEM. ^∗^*P* ≤ 0.05; ^∗∗^*P* ≤ 0.01; ^∗∗∗^*P* ≤ 0.001.

**FIGURE 5:**
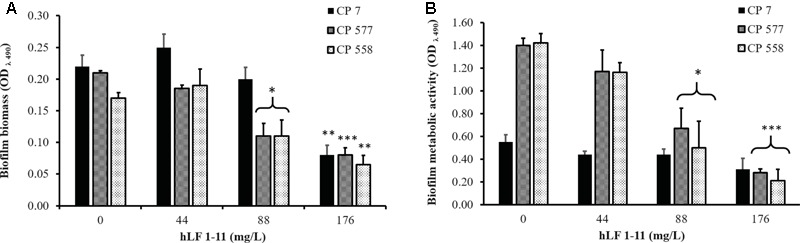
Effect of hLF 1-11 on mature biofilm of *C. parapsilosis.* Yeast cells were incubated for 24 h and then co-incubated with hLF 1-11 at different concentrations for 24 h at 37°C. The peptide anti-biofilm activity was assessed in terms of reduction of biofilm biomass **(A)** and metabolic activity **(B)**. Data are expressed as means of three independent experiments ± SEM. ^∗^*P* ≤ 0.05; ^∗∗^*P* ≤ 0.01; ^∗∗∗^*P* ≤ 0.001.

The inhibitory activity of hLF 1-11 on pre-adherent fungal cells was evaluated on *C. parapsilosis* strain CP 7 (strong biofilm producer). Fungal cells were allowed to adhere to the plastic surface of the microtiter plate for 1.5, 3, 6, and 24 h, respectively. Different peptide concentrations (44 and 88 mg/L) were then added to the cultures and plates were incubated for further 24 h at 37°C. Cells incubated with hLF 1-11 at both concentrations following 1.5 and 3 h of adhesion failed to produce biofilm. Indeed, the peptide induced a complete reduction of biofilm cellular density and metabolic activity (*P* < 0.001) (**Figure 4**). Notably, the activity of hLF 1-11 on the 6 h pre-adherent cells was completely abolished at the lowest peptide concentration used, with a statistically significant anti-biofilm effect observed only at the highest concentration (88 mg/L, **Figure 4**). Furthermore, no peptide activity was detected on preformed mature biofilm (**Figure 4**). The inhibitory activity of hLF 1-11 on preformed biofilm was evaluated also on strains CP 577 and CP 558. The results obtained indicate that hLF 1-11 induced a statistically significant dose-dependent reduction of preformed-biofilm cellular density and metabolic activity at high peptide concentrations only (88 and 176 mg/L) (**Figure 5**). The peptide activity on biofilm formation was similar for all the strains tested, regardless of their fluconazole susceptibility.

### Transcriptional Analysis of *C. parapsilosis* Biofilm-Related Genes

**FIGURE 6:**
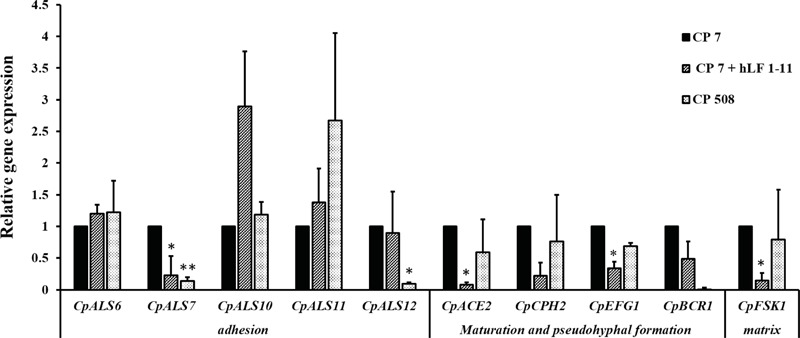
Relative gene expression of *C. parapsilosis* biofilm-related genes assessed by qRT-PCR. Strain CP 7 was co-incubated in the absence and presence of hLF 1-11 44 mg/L for 24 h at 37°C in a 24-wells plate and transcriptional levels of the target genes were determined by qRTPCR using *CpACT1* as reference gene for normalization. Mutant strain CP 508 lacking both copies of *BCR1* was included as control. Data are expressed as means of three independent experiments ± SEM. ^∗^*P* ≤ 0.05; ^∗∗^*P* ≤ 0.01.

In order to understand the molecular basis of hLF 1-11 inhibition of *C. parapsilosis* biofilm, expression of biofilm related genes by qRT-PCR was evaluated following co-incubation of *C. parapsilosis* strain CP 7 with the peptide (44 mg/L) for 24 h at 37°C. Untreated *C. parapsilosis* cells served as control. The results obtained indicated that hLF 1-11 induced a significant reduction of transcriptional levels of *CpALS7, CpACE2, CpEFG1*, and *CpFSK1*, while no significant changes in transcriptional levels of other genes could be observed (**Figure 6**). Transcriptional profile of *BCR1* mutant CP 508 grown in the absence of peptide, showed that two adhesin encoding genes *CpALS7* and *CpALS12* (*CPAR2_404780*) were significantly down regulated (**Figure 6**).

### hLF 1-11 Inhibits *C. parapsilosis* Biofilm Formation on a Catheter Lumen

**FIGURE 7:**
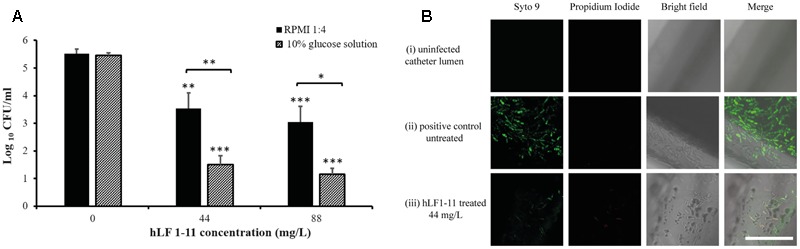
**(A)** Reduction of biofilm formation in catheters co-incubated for 24 h at 37°C with two different concentrations of hLF 1-11, compared with the untreated control. Catheters were incubated in four-fold diluted RPMI or in a 10% glucose solution. Data are expressed as means of three independent experiments ± SEM. ^∗^*P* ≤ 0.05; ^∗∗^*P* ≤ 0.01; ^∗∗∗^ *P* ≤ 0.001. **(B)** CLSM images of (i) un-colonized catheter lumen, and catheters co-incubated with strain CP 7 in the absence (ii) or presence (iii) of 44 mg/L hLF 1-11 for 24 h at 37°C in four-fold diluted RPMI. Bars denote 50 μm.

Considering the ability of *C. parapsilosis* to form biofilm on medical prosthetic materials, the ability of hLF 1-11 to inhibit biofilm formation in an *in vitro* model of catheter-associated *C. parapsilosis* biofilm production was evaluated. Data obtained from colonized catheters (PVC) showed that hLF 1-11 was able to induce a 2-log reduction of sessile cell viability at both the peptide concentrations used (44 mg/L and 88 mg/L) in RPMI diluted in NaPB. It is worth noting that a more pronounced anti-biofilm effect was observed (3.5-log reduction) when a 10% glucose solution was used as experimental condition, in comparison with respective untreated biofilms (**Figure 7**). Furthermore, biofilm formation/inhibition on catheter lumen was monitored by CLSM following fluorescent staining (Syto9). The untreated catheter images (positive control) revealed the presence of Syto9 positive yeast aggregates in a multi-layered biofilm [**Figure 7B**(ii)]. Conversely, hLF 1-11 treated catheters confirmed an almost complete reduction of Syto9 positive yeast cells and the presence of few dead cells stained with propidium iodide [**Figure 7B**(iii)].

### hLF 1-11 Activity on *C. parapsilosis* Mature Biofilm Formed on a Catheter Lumen

**FIGURE 8:**
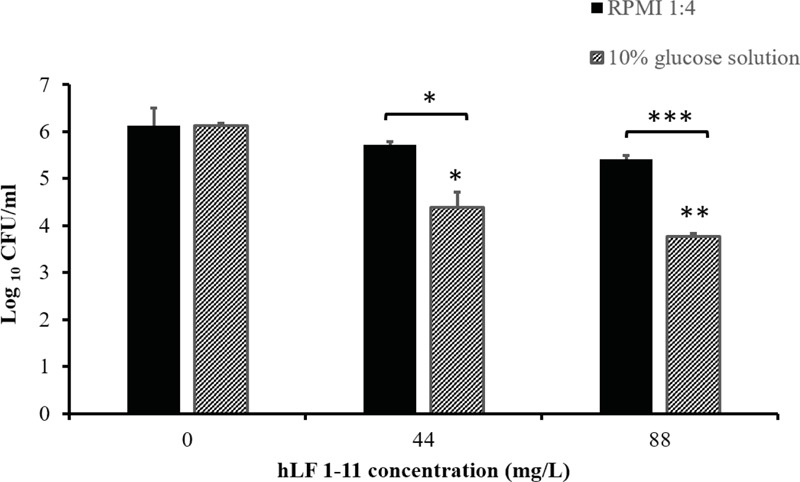
Activity of hLF 1-11 on mature biofilm produced on the catheter lumen following incubation at 37° for 24 h in four-fold diluted RPMI or 10% glucose solution. Data are expressed as means of three independent experiments ± SEM. ^∗^*P* ≤ 0.05; ^∗∗^*P* ≤ 0.01; ^∗∗∗^*P* ≤ 0.001.

Finally, the *in vitro* model of catheter-associated *Candida* biofilm formation was used to evaluate the hLF 1-11 activity on preformed biofilm. Data obtained showed that hLF 1-11 was able to induce approximately 2-log reduction in sessile cell viability at both the peptide concentrations used (44 and 88 mg/L, *P* ≤ 0.05 and *P* ≤ 0.001, respectively) in 10% glucose solution, while no peptide activity was detected on preformed mature biofilm produced on the catheter lumen when RPMI diluted in NaPB was used as biofilm-inducing condition (**Figure 8**).

## Discussion

Three main conclusions can be drawn from the present study, as demonstrated by the following findings. First, the hLF 1-11 peptide induces a drastic reduction in biofilm formation expressed as both biomass and metabolic activity in a dose dependent manner following 24 h co-incubation at 37°C, even at the lowest peptide concentration used. This effect was obtained in all *C. parapsilosis* clinical isolates, which were selected on the basis of their medium to high biofilm production ability. Furthermore, biofilm produced in the peptide absence was characterized by a cellular morphology comprising pseudohyphae and blastoconidia. Upon exposure with hLF 1-11, a complete inhibition of morphogenesis was observed microscopically, with only a few cells predominantly in the yeast form. Kinetic studies were then performed to evaluate whether the peptide activity was exerted mainly at early stages of biofilm formation or it was active also at later time points on the strong biofilm producer isolate (CP 7). Our findings revealed a statistically significant activity on pre-adhered cells for 1.5 and 3 h of incubation at both the hLF 1-11 concentrations used, whereas a significant effect was observed on 6 h pre-adhered cells at the highest concentration only. This finding is in agreement with data recently described for *C. albicans*, where hLF 1-11 significantly altered early stages of biofilm formation, without exerting any effect on mature biofilm (Morici et al., 2016). On medium biofilm producer clinical isolates a significant reduction of both biofilm biomass and metabolic activity could be observed at 88 mg/L hLF 1-11. When exposed to a higher concentration of hLF 1-11 preformed biofilm was significantly reduced in all the clinical isolates tested, suggesting that hLF 1-11 could also be active on *C. parapsilosis* mature biofilm. In a recently described study, hLF 1-11 showed no hemolytic activity (<1%) even at 10× MIC, thus indicating that hLF 1-11 might be safe to be administered at any of the peptide concentrations used in this study (Morici et al., 2017).

Second, a significant reduction in transcriptional level of three biofilm related genes was observed upon co-incubation of *C. parapsilosis* isolate with the peptide under biofilm-inducing conditions as compared to untreated *C. parapsilosis* biofilm. Among the putative *C. parapsilosis* adhesin-encoding genes, *CpALS7* was found to be significantly down-regulated. To date this gene is the only one encoding for an adhesion molecule to have been characterized in *C. parapsilosis* (Bertini et al., 2016)*;* interestingly, it has been recently shown to play a major role in adhesion to biotic surfaces and in the pathogenic potential in a murine model of urinary infection (Bertini et al., 2016). This gene was also down-regulated in the mutant strain lacking both copies of *BCR1* even in the absence of the peptide, suggesting that CpAls7 might be important for adhesion to abiotic surfaces as well. Other *CpALS* genes were not significantly altered upon peptide treatment, nor in the *BCR1* deleted strain, where only *CpALS12* was found to be down-regulated. This result confirms previous data obtained on the *BCR1* mutant, in which the deletion of this important biofilm regulator does not affect *CpALS10* and *CpALS11* transcriptional levels (Ding and Butler, 2007).

One of the genes involved in biofilm maturation and morphogenesis was also found to be reduced by hLF 1-11 exposure in agreement with phenotypic analysis of biofilm formed mainly consisting of isolated yeast cells. This finding parallels previous data obtained with *C. albicans*, showing a strong inhibitory effect on hyphal production by the peptide in this species. However, filamentation in *C. parapsilosis* is mainly due to pseudohyphal formation, since it does not produce true hyphae, and therefore it may play a less significant role in biofilm development and maturation compared to *C. albicans*. Down-regulation of matrix associated *CpFSK1* gene corroborates with experimental data indicating a significant biomass reduction following exposure to hLF 1-11. To better define the role played by the peptide in the regulation of biofilm-associated genes, future studies will be addressed to investigate transcriptional levels of selected genes in response to hLF 1-11 in immature biofilm as well as in later stages of biofilm formation.

Third, hLF 1-11 was able to inhibit biofilm formation in an *in vitro* model of catheter associated *C. parapsilosis* colonization. PCV catheters co-incubated with *C. parapsilosis* Cp7 with and without hLF 1-11 for 24 h revealed a significant inhibitory effect exerted by the peptide in RPMI medium at both the concentrations tested. CLSM imaging confirmed the peptide activity on biofilm formation depicting low number of viable fungal cells on the peptide-treated catheter lumen compared to untreated catheters.

Interestingly, this an anti-biofilm activity was significantly increased when 10% glucose solution was used as biofilm-inducing condition. This finding is clinically relevant considering that 10% glucose solution is often administered via catheters as part of parenteral nutrition in hospitalized patients. This is particularly important in neonatal intensive care units (NICUs) where premature newborns are at high risk of developing catheter related *C. parapsilosis* systemic infections (Trofa et al., 2008; Deepak et al., 2013) Indeed, *C. parapsilosis* represents a major threat for neonates in NICU as it frequently colonizes the hands of health care workers, has high affinity for intravascular devices, and parenteral nutrition (Trofa et al., 2008).

Furthermore, despite the fact that no significant hLF 1-11-anti-biofilm activity was observed on mature biofilm formed in the catheter lumen in RPMI medium, a significant reduction of biofilm cellular density could be detected in mature catheter associated biofilm produced in the presence of 10% glucose solution at both the peptide concentrations used.

The overall findings candidate hLF 1-11 as a promising agent to prevent *C. parapsilosis* and *C. albicans* (Morici et al., 2016) biofilm formation and to treat mature *C. parapsilosis* biofilms grown on PVC catheters used for parenteral nutrition.

Further studies will be aimed at evaluating the hLF 1-11 anti-biofilm activity on biofilms formed by other clinically relevant *Candida* species. Furthermore, the use of hLF 1-11 as coating agent for preventing biofilm formation could also be investigated.

## Ethics Statement

This study was notified to and approved by the local ethical committee, Comitato Etico di Area Vasta Nord-Ovest, University of Pisa, and conducted in full accordance with the principles of the Declaration of Helsinki. Samples were taken as part of the standard patient care. These samples were anonymized by the clinical personnel. Research personnel received and used these samples anonymously. For this type of study, no written informed consent was necessary.

## Author Contributions

AT and AL conceived and designed the experiments. RF, MDL, and CR performed the experiments. DB, PM, AT, AL, and RF analyzed the data. AT and AL drafted the manuscript, with the contribution of MDL, DB, and RF. All authors reviewed and revised the first and final drafts of this manuscript.

## Conflict of Interest Statement

The authors declare that the research was conducted in the absence of any commercial or financial relationships that could be construed as a potential conflict of interest.
