# Supplementary material for: The N-Terminus of Human Lactoferrin Displays Anti-biofilm Activity on Candida parapsilosis in Lumen Catheters
Source: Front Microbiol. 2017 Nov 13;8:2218. doi: 10.3389/fmicb.2017.02218 (PMC5693879; doi:10.3389/fmicb.2017.02218)
Supplement: Supplementary file 1 [file Table_1.DOCX]

| **Target** | **primer forwad 5’🡪 3’** | **primer reverse 5’🡪 3’** |
| --- | --- | --- |
| *CpACT1* | AGTGTGACTTGGATGTCAGAAAGGAATTGT | ACAGAGTATTTTCTTTCTGGTGGAGCA |
| *CpACE2* | TCAGCATAGTCAACAACAGC | TCATCACCTTCATTGAGTTGC |
| *CpCPH2* | TGATACATCTGACTACACTTCGC | GTCTTCGGCACCAGCG |
| *CpEFG1* | GTTCATACTATCAAGGTATGGAGGAGC | GGTATTGGTATGGTAAGACGA |
| *CpFSK1* | CACGCTGACTACATTGGAGGT | CAGGAGTCAAGGTGTTCATCTT |
| *CpALS7* | TCGAGTTCCTAATGGTGCAG | CCTTCTTCACCCCAGTTTTG |
| *CpALS6* | CGGCGCAGATGTGCTAATG | AAAGTCACCACCACCGAGG |
| *CPAR2_500660 (ALS 10)* | GGGATCAGCAAATTCTGTCGA | CCAGCGGTAAAACATTGGGA |
| *CPAR2_404770 (ALS 11)* | TGTCCTCGACAACTCCAGCTT | GGTTCTAAAATGGGTGGAATG |
| *CPAR2_404780 (ALS 12)* | AACGTCCAACAGGTCAAGTG | CTCCCCATTTATTGATTGTG |

**Table S1.** Sequences of primers used for qRT-PCR
