# Supplementary figures and images for: The N-Terminus of Human Lactoferrin Displays Anti-biofilm Activity on Candida parapsilosis in Lumen Catheters
Source: Front Microbiol. 2017 Nov 13;8:2218. doi: 10.3389/fmicb.2017.02218 (PMC5693879; doi:10.3389/fmicb.2017.02218)

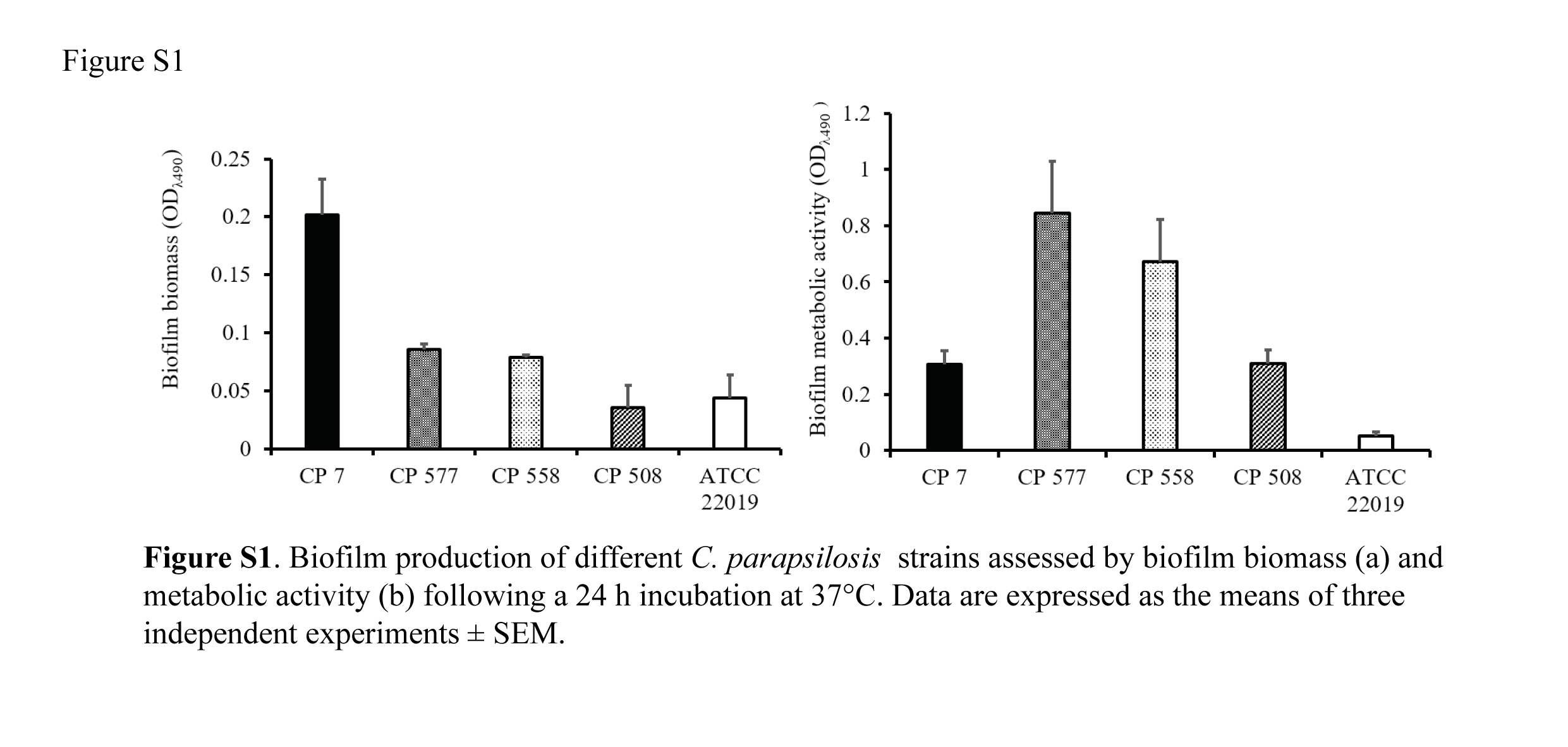

Supplement: Supplementary file 2 [file Image_1.TIF]
